# Evaluation of Levels of Triamcinolone Acetonide in Human Perilymph and Plasma After Intratympanic Application in Patients Receiving Cochlear Implants: A Randomized Clinical Trial

**DOI:** 10.1001/jamaoto.2021.2492

**Published:** 2021-09-30

**Authors:** Valerie Dahm, Julia Clara Gausterer, Alice Barbara Auinger, Clemens Honeder, Franz Gabor, Gottfried Reznicek, Alexandra Kaider, Dominik Riss, Christoph Arnoldner

**Affiliations:** 1 Department of Otorhinolaryngology–Head and Neck Surgery, Medical University of Vienna, Vienna, Austria; 2 Department of Pharmaceutical Technology and Biopharmaceutics, University of Vienna, Austria; 3 Department of Pharmacognosy, University of Vienna, Vienna, Austria; 4 Center for Medical Statistics, Informatics, and Intelligent Systems, Medical University of Vienna, Vienna, Austria

## Abstract

**Importance:**

The use of intratympanically applied steroids is of increasing interest. Consequently, research has focused on finding an ideal drug that diffuses through the round window membrane and can be retained in the perilymph.

**Objective:**

To compare levels of triamcinolone acetonide (TAC) in perilymph and plasma after intratympanic injection.

**Design, Setting, and Participants:**

This randomized clinical trial included 40 patients receiving cochlear implants at a single tertiary care center in Vienna, Austria. Patients were randomized to 1 of 4 treatment groups receiving 1 of 2 intratympanic doses of TAC (10 mg/mL or 40 mg/mL) at 1 of 2 approximate time points (24 hours or 1 hour) before sampling the perilymph. Inclusion was carried out between November 2017 and January 2020, and data were analyzed in December 2020.

**Interventions:**

All patients underwent intratympanic injection of TAC. During cochlear implantation, perilymph and plasma were sampled for further analysis.

**Main Outcomes and Measures:**

Levels of TAC measured in perilymph and plasma.

**Results:**

Among the 37 patients (median [range] age, 57 [26-88] years; 18 [49%] men) included in the analysis, TAC was present at a median (range) level of 796.0 (46.4-7706.7) ng/mL. In the majority of patients (n = 29; 78%), no drug was detectable in the plasma after intratympanic injection. Levels above the limit of detection were less than 2.5 ng/mL. The 1-factorial analysis of variance model showed lower TAC levels in the group that received TAC, 10 mg/mL, 24 hours before surgery (median, 271 ng/mL) compared with the group that received TAC, 10 mg/mL, 1 hour before surgery (median, 2877 ng/mL), as well as in comparison with the groups receiving TAC, 40 mg/mL, 24 hours before surgery (median, 2150 ng/mL) and 1 hour before surgery (median, 939 ng/mL). The 2-factorial analysis of variance model showed lower TAC levels in the group receiving TAC, 10 mg/mL, 24 hours before surgery than the group receiving TAC, 10 mg/mL, 1 hour before surgery, and higher TAC levels in the group receiving TAC, 40 mg/mL, 24 hours before surgery compared with the group receiving TAC, 10 mg/mL, 24 hours before surgery. Patients with thickening of the middle ear had statistically significantly higher plasma levels (median, 1.4 ng/mL vs 0 ng/mL) and lower perilymph levels (median, 213.1 ng/mL vs 904 ng/mL) than individuals with unremarkable middle ear mucosa.

**Conclusions and Relevance:**

In this randomized clinical trial, TAC was shown to be a promising drug for intratympanic therapies, with similar levels in perilymph 1 hour and 24 hours after injection (distinctly in the groups receiving the 40 mg/mL dose). There was also minimal dissemination to the plasma, especially in patients with unremarkable middle ear mucosa.

**Trial Registration:**

ClinicalTrials.gov Identifier: NCT03248856

## Key Points

**Question:**

How much intratympanically injected triamcinolone acetonide diffuses to the perilymph and plasma?

**Findings:**

In this randomized clinical trial of 40 patients undergoing cochlear implantation, there were similar levels of triamcinolone acetonide in the perilymph 1 hour and 24 hours after injection, and triamcinolone acetonide could be quantified in all analyzed patients at a median (range) level of 796.0 (46.4-7706.7) ng/mL. There was minimal dissemination to the plasma, especially in patients with unremarkable middle ear mucosa.

**Meaning:**

Triamcinolone acetonide can be found in the perilymph after intratympanic application, even on the day after injection.

## Teaser

This randomized clinical trial compares levels of triamcinolone acetonide in perilymph and plasma after intratympanic injection.

## Introduction

Many inner ear diseases are treated with systemic corticosteroids. However, owing to minimal blood flow to the cochlea and the blood-perilymph barrier,^1,2^ only a small proportion of intravenously applied prednisolone reaches the perilymphatic fluid.^3,4^ Studies investigating glucocorticoid absorption and distribution have mainly been conducted in animals. Bird et al^5,6^ investigated perilymph concentrations 0.5 to 3 hours after intratympanic (IT) application of methylprednisolone or dexamethasone in humans. Concentrations of methylprednisolone and dexamethasone were 33- to 260-fold higher after IT than after intravenous application depending on the dose and drug.^5,6^ Application via a transtympanic approach has received increasing interest in recent years. Drugs, however, have been optimized for intravenous, intradermal, or intramuscular application and absorption but lack adaptation for this new treatment method.^7^ The goal of this randomized clinical trial was to investigate and compare perilymph and plasma concentrations of triamcinolone acetonide (TAC) after IT application, which, to the best of our knowledge, has not been previously evaluated in humans.

## Methods

### Study Population

Each patient provided written informed consent to participate in this prospective study, which was approved by the ethics committee of the Medical University of Vienna. Inclusion criteria were that patients were between 18 and 90 years old, undergoing cochlear implantation, and willing to participate in the study. Exclusion criteria were that patients were younger than 18 years, were receiving steroids on a regular basis or had received steroids intravenously or orally preoperatively, had contraindications against the administration of TAC, or had contraindications against IT injections (Supplement 1).

**Figure 1.**
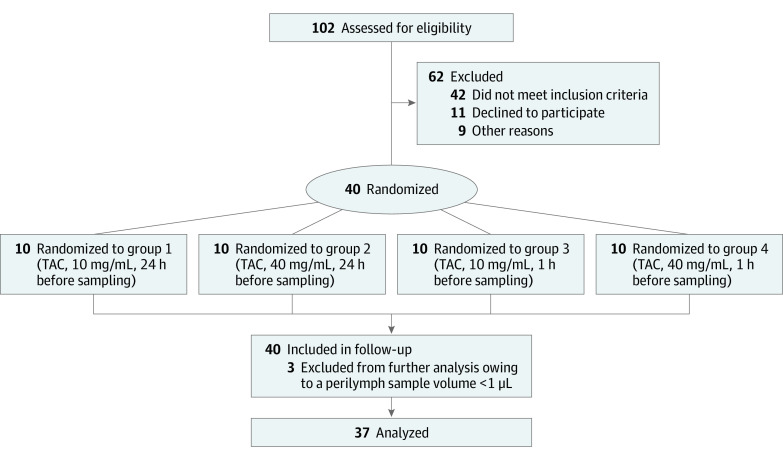
CONSORT Diagram TAC indicates triamcinolone acetonide.

Forty patients were randomly assigned to 1 of 4 groups. Groups 1 and 2 received IT TAC approximately 24 hours before surgery, whereas groups 3 and 4 received the injection approximately 1 hour before surgery (Table 1). Groups 1 and 3 were treated with TAC, 10 mg/mL, and groups 2 and 4 with TAC, 40 mg/mL (Figure 1). Causes of hearing loss were distributed as follows: otosclerosis (n = 1), Meniere disease (n = 2), labyrinthitis (n = 1), progressive hearing loss after cytomegalovirus infection (n = 1), progressive sensorineural hearing loss (n = 24), sudden sensorineural hearing loss (n = 5), and vestibular schwannoma (n = 3).

**Table 1.** Characteristics of the 4 Different Groups and the Timing of Intratympanic TAC Application, Dose, and Volume^a^

Characteristic	Median (range)				*P* value^b^
	Group 1	Group 2	Group 3	Group 4	
No. of patients	9	10	9	9	NA
TAC dose, mg/mL	10	40	10	40	NA
Age, y	57 (47-82)	62 (46-78)	41 (26-68)	64 (40-88)	.004
Injection volume, mL	0.65 (0.30-1.00)	0.60 (0.20-1.00)	0.40 (0.35-0.90)	0.40 (0.15-1.00)	.55
BMI	25.4 (19.0-29.3)	29.6 (24.1-50.0)	28.4 (21.8-41.3)	27.0 (22.5-31.8)	.10
Actual time between injection and sample					
Hours	21.6 (19.0-25.8)	20.9 (14.6-25.1)	1.7 (1.0-2.2)	1.6 (0.9-2.2)	<.001
Minutes	1297 (1140-1545)	1255 (875-1505)	104 (58-130)	96 (51-130)	

Abbreviations: BMI, body mass index, calculated as weight in kilograms divided by height in meters squared; NA, not applicable; TAC, triamcinolone acetonide.

^a^ Three patients had to be excluded owing to a sample volume less than 1 μL.

^b^ For group comparison, *P* values were calculated using a 1-factorial analysis of variance model.

### Intratympanic Application of Triamcinolone Acetonide

On the day before cochlear implantation, patients were placed in a supine position. Xylocaine spray, 10 mg/puff, was used as local anesthesia. The suspension (TAC) was applied using a 25-gauge (0.50 × 90 mm, 3.50 in) needle through the tympanic membrane. Patients were asked to stay in the position for 20 minutes and instructed not to speak or swallow.

Patients receiving TAC on the day of surgery were administered general anesthesia and positioned for cochlear implantation. The injection was then performed as previously described. The patient’s position was not altered after the injection. Patients were blinded to the dose given, while physicians were not.

### Surgery and Perilymph Sampling

In all 40 patients, cochlear implantation was carried out via a standard mastoidectomy and facial recess approach, during which irrigation was used. The bony overhang of the round window was reduced with no irrigation to avoid dilution effect of the perilymph sample. The intact round window membrane was perforated with a sterile disposable aspirator (MediPlast [Curad], 0.4 x 70 mm, 27 gauge). The aspirated perilymph (approximately 20 μL) was immediately transferred to a sterile 0.2-mL safe lock tube and stored at −80 °C. In 3 patients, less than 1 μL could be sampled, which is why no further analysis was possible.

### Blood Sample

Simultaneously with aspiration of perilymph, a blood sample was taken in the standard fashion. Blood was drawn in a heparin tube (9 mL). The vacutainer was gently swiveled 5 times. The sample was then centrifuged for 20 minutes at 20 °C with 2000 relative centrifugal force. Plasma was transferred to a 2-mL safe lock tube and stored at −80 °C.

### Triamcinolone Acetonide

In this study, patients received IT TAC (Volon A [Dermapharm GmbH]) containing either 40 mg/mL or 10 mg/mL of TAC. In preliminary experiments, the particle-size distribution of TAC was determined in 0.9% sodium chloride/0.1% polysorbate 80 by laser-light scattering using a Mastersizer 3000 (Malvern Instruments). Triamcinolone acetonide, 10 mg/mL, had a pH of 6.27 and an osmolality of 318 mOsm/kg; 90% of particles had a mean (SD) particle size less than 23.8 (1.0) μm, 50% of particles were below and above 12.8 (0.9) μm, and 10% of the particle population were less than 5.6 (0.8) μm. Triamcinolone acetonide, 40 mg/mL, had a pH of 6.19 and an osmolality of 331 mOsm/kg. The mean (SD) size of 90% of particles was less than 22.1 (1.6) μm, 50% of particles were below and above 11.6 (1.4) μm, and 10% of the particle population were less than 4.8 (1.1) μm. Both formulations contained 9.9 mg of benzyl alcohol per 1 mL, as well as sodium carboxymethyl cellulose, polysorbate 80, sodium chloride, and water.

### Sample Preparation

Perilymph samples were prepared by diluting a definite volume of perilymph with ice-cold methanol to obtain a total minimum volume of 20 μL to allow for high-performance liquid chromatography–mass spectrometry analysis in triplicate (dilution range, 1:4 to 1:21). Traces of protein precipitates were removed by centrifugation, and the supernatants were collected and stored at 4 °C. For preparation of plasma samples, 100 μL of plasma was precipitated by the addition of 300 μL of ice-cold methanol, followed by centrifugation for 10 minutes at 20 800 relative centrifugal force and 4 °C. Plasma was collected and stored at 4 °C. All samples were kept on ice during sample preparation.

### Quantification of Triamcinolone Acetonide

Triamcinolone acetonide was quantified by high-performance liquid chromatography. The samples were analyzed using liquid chromatography–mass spectrometry on an UltiMate 3000 RSLC series system (Thermo Fisher Scientific) coupled to a triple quadrupole mass spectrometer (API 4000 [AB Sciex]) equipped with an orthogonal electrospray ionization source operated in positive mode.

Liquid chromatography separation was performed on an Acclaim RSLC 120 C18 column (3 μm, 100 × 2.1 mm; Thermo Fisher Scientific) preceded by an Acclaim 120 C18 guard cartridge (5 μm, 10 × 2 mm; Thermo Fisher Scientific) at a flow rate of 0.5 mL/min and a column temperature of 25 °C. The mobile phase consisted of a linear gradient mixed from 0.1% aqueous formic acid (mobile phase A) and acetonitrile (mobile phase B). The gradient ranged from 20% mobile phase B at 0 minutes to 95% mobile phase B in 5 minutes, purging with 95% mobile phase B for 1 minute, then 20% mobile phase B to equilibrate the column for 4 minutes before applying the next sample (total analysis time, 10 minutes). Triamcinolone acetonide eluted at 4.13 minutes. Triamcinolone acetonide was selectively and sensitively detected and quantified using tandem mass spectrometry fragmentation of TAC, giving a quasimolecular ion at mass-to-charge ratio (m/z) of 435.4 [M+H]^+^. Multiple reaction monitoring m/z 435.4/415.0 (quantifier) and m/z 435.4/213.1 (qualifier) were used for calibration curves with external standard TAC (injection volume, 5 μL) to obtain a linear concentration range from 0.1 to 5000 ng/mL (y = 559x + 3050; correlation coefficient, 0.9986). The lower limit of detection was 0.1 ng/mL, and the lower limit of quantification was set at 0.5 ng/mL. The triple quadrupole mass spectrometer operated with the following parameters: ESI pos, IS 5500, EP 10, CUR 10, GS1 40, GS2 40, TEM 500 °C, CAD 4, CEM 3000, DF −100. MRM m/z 435.4/415.0: DP 66, CE 11, CXP 26; MRM, m/z 435.4/213.1: DP 81, CE 37, CXP 34; dwell time for each MRM150 ms (ESI indicates electrospray ionization; IS, ion spray voltage; EP, entrance potential; CUR, curtain gas; GS1, ion source gas 1; GS2, ion source gas 2; TEM, temperature; CAD, collisionally activated dissociation; CEM, channel electron multiplier; DF, deflector; MRM, multiple reaction monitoring; DP, declustering potential; CE, collision energy; CXP, collision cell exit potential).

### Sample-Size Calculation

The primary aim of the study was to determine and compare perilymph and plasma levels of IT-injected TAC. The required sample size was calculated based on the paired *t* test and a 2-sided significance level of 5%. Perilymph and blood concentrations of dexamethasone are known from the literature in 12 patients,^5^ and log10-transformed values were used for calculations owing to the skew distribution. Retransforming the mean log values to the original scale resulted in geometric mean values of 1.26 mg/L for perilymph levels and 0.001 mg/L for plasma levels. The SD of the paired differences of the log10-transformed values was calculated as 0.81. To detect an effect size of 0.5 SDs, which is a difference of 0.4 on the log scale (a difference in the geometric means of 1.26-0.50) with a statistical power of 86%, a total of 40 patients needed to be included in the study (nQuery Advisor, version 7.0 [Statistical Solutions]). The primary study aim was to determine and compare perilymph and plasma levels of IT-applied TAC; therefore, sample-size calculation was based on this objective. Additionally, the comparison of different doses and sampling schemes is an unanswered question, so we decided to randomize the sample in the 4 different groups. The power to detect relevant differences between the groups is small.

### Statistical Analysis

Median values with ranges (presented as minimum to maximum) are given for continuous variables. The nonparametric Wilcoxon signed rank test was used to compare TAC levels in perilymph and plasma. Analysis of variance (ANOVA) models were used for group comparisons of the TAC levels in perilymph. First, groups 1 to 4 were compared using a 1-factorial ANOVA design. The Tukey-Kramer method was used for multiplicity-adjusted pairwise group comparisons. Second, two 1-factorial ANOVA models were applied, testing for the overall dose (10 mg/mL vs 40 mg/mL) and the overall time point (1 hour vs 24 hours) effects. Third, a 2-factorial ANOVA model was used, including the 2 factors of dose and time between application and sampling, together with an interaction term to test for a dose-dependent time effect and a time-dependent dose effect, respectively. Due to the skew distribution of the TAC levels in perilymph, log10-transfomed values were used as dependent variables for all ANOVA and regression models. To quantify the strength of the effects on TAC levels, the geometric mean ratios (GMRs) with 95% CIs were determined by retransforming mean differences from the logarithmic to the original scale. To compare the TAC levels in patients with an aerated middle ear to patients with mucosal disease, the 2-sample *t* test was used for perilymph (log10 transformed) and the exact Wilcoxon rank sum test for plasma. Analysis was conducted using SAS statistical software, version 9.4 (SAS Institute), and a 2-sided *P* < .05 was considered statistically significant.

## Results

### Triamcinolone Acetonide Levels in Perilymph

In this study, 40 patients undergoing cochlear implantation received IT TAC. In 3 patients, the perilymph sample volume was less than 1 μL, which was not sufficient for further analysis. The remaining 37 patients (median [range] age, 57 [26-88] years; 18 [49%] men) had perilymph samples with a quantifiable concentration of TAC. The median (range) TAC level in the perilymph was 796.0 (46.4-7706.7) ng/mL.

### Triamcinolone Acetonide Levels in Plasma

Triamcinolone acetonide concentrations in the plasma were lower than the lower limit of detection in 29 of 37 patients. The median (range) plasma TAC level in the remaining 8 patients was 1.7 (1.1-2.5) ng/mL. The median (range) TAC level in the plasma for all patients was 0 (0-2.4) ng/mL. All 8 patients with detectable levels of TAC in the plasma were in 1 of the 2 groups receiving TAC, 10 mg/mL. The TAC levels in perilymph were a median (range) of 796.0 (44.8-7705.4) ng/mL higher than in plasma.

### Effect of Triamcinolone Acetonide Dose and Time Point

The median (range) TAC concentrations in perilymph in the 4 different treatment groups were 271.0 (46.4-670.7) ng/mL in group 1; 2149.7 (51.9-5916.0) ng/mL in group 2; 2877.3 (396.4-7706.7) ng/mL in group 3; and 939.0 (329.4-4641.0) ng/mL in group 4. The 1-factorial ANOVA model showed that the difference between the 4 groups was statistically significant: group 1 had statistically significantly lower TAC levels in perilymph than the other 3 groups (GMR: group 1 vs group 2, 0.15 [95% CI, 0.04-0.62]; group 1 vs group 3, 0.12 [95% CI, 0.03-0.51]; and group 1 vs group 4, 0.22 [95% CI, 0.05-0.95]). There were no statistically significant differences between any other 2 groups (GMR: group 2 vs group 3, 0.79 [95% CI, 0.19-3.25]; group 2 vs group 4, 1.47 [95% CI, 0.36-6.04]; and group 3 vs group 4, 1.86 [95% CI, 0.44-7.91]).

The median (range) TAC level in perilymph in the 2 groups given TAC, 10 mg/mL, was 440.0 (46.4-7706.7) ng/mL and in the 2 groups given TAC, 40 mg/mL, was 1046.1 (51.9-5916.0) ng/mL. The 1-factorial ANOVA model testing for the overall dose (10 mg/mL vs 40 mg/mL) effect showed that the comparison was not statistically significant (GMR, 0.52 [95% CI, 0.21-1.29]).

**Figure 2.**
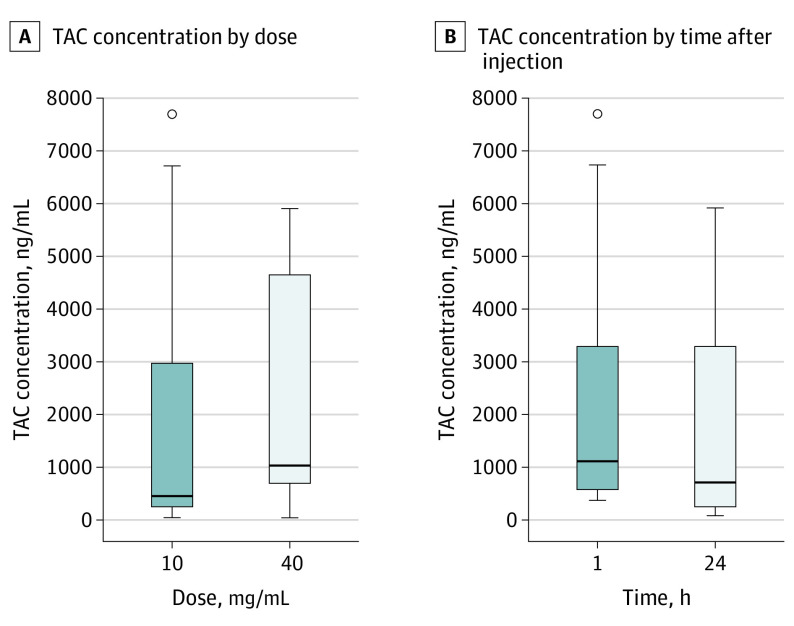
Box Plots of Triamcinolone Acetonide (TAC) Concentrations in Perilymph The horizontal line in the boxes indicates the median. The boundaries of the boxes represent the lower and upper quartiles. The whiskers are drawn from the edge of the boxes to the largest and smallest values that are outside of the boxes but within 1.5 SDs. Circles depict outliers.

The median (range) TAC levels in perilymph were 670.7 (46.4-5916.0) ng/mL in groups 1 and 2 (TAC given 24 hours before surgery) and 1086.0 (329.4-7706.7) ng/mL in groups 3 and 4 (TAC given 1 hour before surgery). The difference between the time points of the groups was not statistically significant (GMR, 0.44 [95% CI, 0.18-1.07]; Figure 2).

**Figure 3.**
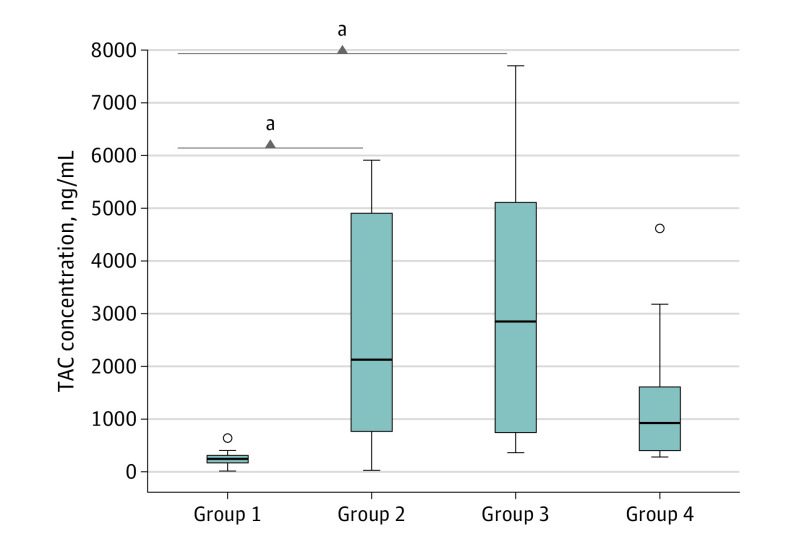
Box Plot of Triamcinolone Acetonide (TAC) Concentrations in the Perilymph Among the 4 Treatment Groups The horizontal line in the boxes indicates the median. The boundaries of the boxes represent the lower and upper quartiles. The whiskers are drawn from the edge of the boxes to the largest and smallest values that are outside of the box but within 1.5 SDs. Circles depict outliers. Group 1 was given TAC, 10 mg/mL, 24 hours before surgery; group 2, TAC, 40 mg/mL, 24 hours before surgery; group 3, TAC, 10 mg/mL, 1 hour before surgery; group 4, TAC, 40 mg/mL, 1 hour before surgery. ^a^Denotes statistical significance according to the 2-factorial analysis of variance model.

A 2-factorial ANOVA model was used, including the 2 factors of dose and time between application and sampling. Within the groups that received the lower dose, the TAC concentration was statistically significantly lower after 24 hours than after 1 hour (GMR, 0.12 [95% CI, 0.04-0.36]). There was no statistically significant difference between the groups receiving 40 mg/mL (GMR, 1.47 [95% CI, 0.51-4.26]). On the other hand, TAC levels in the perilymph of patients who underwent sampling 1 hour after IT injection were not statistically significantly different between dose groups (GMR, 0.54 [95% CI, 0.18-1.60]). However, TAC concentrations in the perilymph were statistically significantly higher in the group receiving the 40 mg/mL dose than the group receiving the 10 mg/mL dose when TAC was injected 24 hours before the surgery (GMR, 6.63 [95% CI, 2.29-19.16]). The results of all 4 groups according to time point and dose are depicted in Figure 3.

### Chronic Middle Ear Disease

In 4 patients, the middle ear was filled with scar tissue and/or thickened mucosa (Table 2). The TAC levels in perilymph for these patients were 46.4, 96.8, 396.4, and 329.4 ng/mL. The median (range) perilymph TAC level in these patients was 213.1 (46.4-396.4) ng/mL compared with 904.0 (51.9-7706.7) ng/mL in the remaining 33 patients with a healthy middle ear. The difference was statistically significant (GMR, 0.14 [95% CI, 0.04-0.52]). The TAC levels in plasma were statistically significantly higher in the group with mucosal disease (median [range], 1.4 [0-2.5] ng/mL) than in the patients with an unremarkable middle ear (median [range], 0 [0-2.3] ng/mL).

**Table 2.** Triamcinolone Acetonide (TAC) Values for the 4 Patients With Mucosal Disease

Patient	Group	TAC, ng/mL	
		Perilymph	Plasma
1	1	46.4	1.6
2	1	96.8	1.1
3	3	396.4	2.5
4	4	329.4	ND

Abbreviation: ND, not detectable.

### Injected Volume

The median (range) injected volume across all groups was 0.50 (0.2-1.0) mL (Table 1). The distribution of TAC levels in perilymph, when compared with a given dose of TAC, did not show a correlation (*r* = 0.187).

## Discussion

To our knowledge, this is the first study in humans in which IT-injected TAC was measured in the perilymph, as well as its distribution to the plasma. Comparable with previous studies on other glucocorticoids, the range of drug concentrations in the perilymph varies substantially.^5,6^

In the present study, 4 patients had some form of middle ear pathology with scaring and/or thickened mucosa, which lead to lower TAC levels in the perilymph and higher TAC levels in plasma. Chronic middle ear disease can lead to thickening of the middle ear mucosa, as well as angiogenesis. Two of the 4 patients with middle ear pathologies were in group 1, 1 was in group 3, and 1 in group 4. The influence of chronic middle ear disease on the perilymph and plasma levels of an intratympanically applied drug has, to the best of our knowledge, not been shown thus far and obviously has important implications for clinical routine. First, levels of a substance in the perilymph after IT application will not reach the same levels as in patients with a healthy middle ear. In addition, the distribution of drugs to the plasma may be increased in patients with mucosal disease. It is important to note that these results have only been shown in a small sample size in the present study and need to be confirmed in larger series.

The main advantage of IT application is the avoidance of systemic adverse effects. This study could prove that local application leads to a considerably higher TAC level in the perilymph than the plasma. Although serial samples were not and could not be performed, TAC levels in the perilymph were not statistically significantly lower in the group that received TAC 24 hours before surgery compared with the group that received TAC 1 hour before surgery, which may be an indication of the suspected depot effect occurring due to the crystalline suspension of TAC, which dissolves slowly.^8,9,10^ To evaluate a possible interaction effect between the dose and delay between IT injection and sampling, a 2-factorial ANOVA model was applied and showed a statistically significant interaction. In the group that received TAC, 10 mg/mL, 1 hour before surgery, TAC levels were statistically significantly higher. The group receiving TAC, 40 mg/mL, 24 hours before surgery had statistically significantly higher TAC levels. Although containing different concentrations of TAC, the solubility of TAC in both formulations (10 mg/mL vs 40 mg/mL) is equally limited by the molecule’s lipophilic nature, meaning that the amount of dissolved TAC is the same regardless of the formulation and that both formulations represent supersaturated solutions. Without knowledge of the exact composition of the formulations (eg, sodium chloride, polysorbate 80), the 13 mM higher osmolality in the TAC, 40 mg/mL, formulation cannot be attributed to the presence of a higher concentration of dissolved TAC. The higher intracochlear TAC levels in perilymph after 24 hours could be because of the 40-mg/mL dose containing 75% more TAC than the 10-mg/mL dose, resulting in an increased viscosity and probably improving adhesion to the round window membrane and limiting drainage via the eustachian tube, which are both required for successful intratympanic drug delivery to the inner ear. This effect on viscosity is further pronounced by the presence of sodium carboxymethylcellulose, which probably contributes to prolonged contact between the dissolved TAC fraction and the round window membrane.

Previous studies led to the assumption that longer drug maintenance in the basal region of the cochlea leads to a higher chance of extending into apical regions.^7^ The TAC suspension used in the present study is combined with benzyl alcohol, which has been shown to increase round window membrane permeability by a factor of 3 to 5.^11^ Benzyl alcohol also keeps substances from being cleared from the middle ear space as quickly,^12^ which may also enhance the suspected depot effect of the crystalline suspension of TAC.

It remains unclear if any active transporters increase or decrease perilymph levels of certain drugs. Salt and Plontke^7^ recently conducted a simulation study showing that TAC has favorable pharmacological properties compared with steroids, which are mainly used for IT application. Triamcinolone acetonide was proposed as a promising IT steroid owing to the high permeability of the round window membrane and the long half-life of its metabolite triamcinolone. However, in a more recent study in guinea pigs, as well as a simulation of sequential sample data, Salt et al^10^ supported the use of triamcinolone instead of TAC because it is retained in the perilymph longer and may result in improved distribution to the cochlear apex. In that study, only 2 guinea pigs were treated with a somewhat comparable setup as the present study; a TAC solution (as opposed to a loaded gel) was applied to the round window, circumventing absorption through the thin otic capsule. The perilymph was sampled 1 hour later and contained 710 ng/mL of TAC. Loading and sampling of the lateral semicircular canal were measured to determine how much TAC was retained in the perilymph. The results showed an extremely high elimination rate, which was calculated by simulation (elimination half-time was 12 minutes in the scala tympani and 34 minutes in the scala vestibuli). Although serial sampling could not be performed, overall, the present results show that TAC levels were similar 1 hour and 24 hours after IT application, which means that either the elimination is much slower than calculated previously^10^ or the remaining solution continuously diffuses through the round window membrane. Honeder et al^8^ reported a TAC half-life of 44.9 hours when the drug was applied intratympanically with a poloxamer 407 hydrogel.

It is important to note that this study’s primary aim was to determine and compare IT-applied TAC levels in perilymph and plasma; therefore, sample size calculation was based on this objective. Additionally, the comparison of different doses and sampling schemes is an unanswered question, so we decided to randomize the sample into the 4 different groups. Of course, the power to detect relevant differences between the groups is small, and an additional study powered for this comparison is needed.

### Limitations

One of the limitations of the current study is the small sample size of each group. A further important topic is that physicians applying IT TAC were not blinded to the doses, which might have affected the dose given. Sampling of perilymph was carried out during cochlear implantation. During mastoidectomy and the facial recess approach, irrigation is used, which might influence TAC levels in perilymph.

## Conclusions

Results of this randomized clinical trial showed that TAC is a promising drug for intratympanic therapies with similar levels in perilymph 1 hour and 24 hours after injection (distinctly in the group receiving TAC, 40 mg/mL). Additionally, it leads to minimal dissemination to the plasma, especially in patients with unremarkable middle ear mucosa. Therefore, TAC remains an important drug for treating inner ear disorders via IT injection and should be evaluated further in future studies.

## Notes

Supplement 1. Trial Protocol

Supplement 2. Data Sharing Statement
