# Supplementary material for: Evaluation of Levels of Triamcinolone Acetonide in Human Perilymph and Plasma After Intratympanic Application in Patients Receiving Cochlear Implants: A Randomized Clinical Trial
Source: JAMA Otolaryngol Head Neck Surg. 2021 Sep 30;147(11):1–7. doi: 10.1001/jamaoto.2021.2492 (PMC8485207; doi:10.1001/jamaoto.2021.2492)
Supplement: Supplement 1. — Trial Protocol [file jamaotolaryngolheadnecksurg-e212492-s001.pdf]

KOORDINATIONSZENTRUM
FÜR KLINISCHE STUDIEN
MEDIZINISCHE UNIVERSITÄT WIEN

CLINICAL STUDY PROTOCOL

Triamcinolone levels in cochlear perilymph

Triamcinolone levels

Version 1.0 / Date 28.07.2017

Project number: 1456/2017

Confidentiality Statement
The information contained in this document, especially unpublished data, is the property of the sponsor of this study. It is therefore provided to you in confidence as an Investigator, potential Investigator, or consultant, for review by you, your staff, and an Independent Ethics Committee or Institutional Review Board. It is understood that this information will not be disclosed to others without written authorization from the sponsor or the study personnel except to the extent necessary to obtain informed consent from those persons to whom the study drug may be administered.

**Test drug (IMP) and Pharmaceutical Company**	Triamcinolone acetonide (Volon® A crystal suspension)<br><br>Dermapharm GmbH
**Protocol author**	Dr. Valerie Dahm
**Investigator**	Assoc. Prof. PD Dr. Christoph Arnoldner
**Document type**	Clinical study protocol
**Study phase**	Phase I
**Document status**	Final
**Date**	28.07.2017
**Number of pages**	38

1. SPONSOR, INVESTIGATOR, MONITOR AND SIGNATURES

Sponsor/or representative (OEL) (AMG §§ 2a, 31,32)

Univ. Prof. Dr. Wolfgang Gstöttner, Department of Otorhinolaryngology
Medical University of Vienna, Austria

________________ ___________

Signature (OEL) Date

Investigator (AMG §§ 2a, 35,36)

Assoc. Prof. PD Dr. Christoph Arnoldner, Department of Otorhinolaryngology
Medical University of Vienna, Austria

_________________ ___________

Signature Date

Monitor/ or Representative of CRO (AMG §§ 2a, 33,34)

DI Rudolfs Liepins
Study coordinator Department of Otorhinolaryngology
Medical University of Vienna, Austria

_________________ ___________

Signature Date

Clinical Trials Centers:

Department of Otorhinolaryngology Medical University of Vienna, Austria

Associated Departments

Department of Pharmaceutical Technology & Biopharmaceutics, University of Vienna, Austria
Mag. Pharm Julia Clara Gausterer
a.o. Univ. Prof. Dr. Franz Gabor

2. PROTOCOL SYNOPSIS

TITLE	Triamcinolone levels in cochlear perilymph - a prospective, randomized clinical trial
OBJECTIVES	**Primary Objective**<br>• *Demonstrate absorption of Triamcinolone acetonide in cochlear perilymph in comparison to dissemination to the blood circulation*<br>**Secondary Objectives**<br>• *Assess the stability of triamcinolone acetonide levels in the cochlear perilymph*<br>• *Assess perilymph concentrations and blood concentrations of triamcinolone acetonide after administration of different Triamcinolone acetonide doses.*
DESIGN / PHASE	*Prospective phase I study.*
STUDY PLANNED DURATION	**First patient First visit**: *4Q* 2017; **Last patient First visit**: *3Q* 2019; **Last patient Last visit**: *4Q* 2019
CENTER(S)<br>/ COUNTRY(IES)	*One center.*<br>*Austria*
PATIENTS / GROUPS	*10 patients/group*<br>*4 groups:*<br>*Group 1:*<br>Volon® *10mg - sampling 24h after administration*<br>*Group 2*<br>Volon® *40mg - sampling 24h after administration*<br>*Group 3*<br>Volon® *10mg - sampling 1h after administration*<br>*Group 4*<br>Volon® *40mg - sampling 1h after administration*<br><br>*Group 5 –* Volon® *40mg – sampling 24h after administration - RWM, SCC and CSF*<br><br>*1:1:1:1 (Group 1-4)*
INCLUSION CRITERIA	• *Patients between 18 and 90 years will be included in the study, who will undergo a cochlear implantation and are willing to participate in the study*
EXCLUSION CRITERIA	• *Patients younger than 18 years*<br>• *Patients who receive cortisone on a regular basis or receive cortisone i.v. or p.o. preoperatively*<br>• *Patients with contraindications against the administration of* ***Volon®*** A
STUDY PERIODS	• *Patients will be included in the study over a time period of two years. The active study phase of each patient will be a maximum of nine days.*
INVESTIGATIONAL DRUG	***Triamcinolone acetonide (Volon® A crystal suspension) 40mg***<br>*Dose: 1ml*

	Triamcinolone acetonide (Volon® A crystal suspension) 10mg<br>*Dose: 1ml*
COMPARATIVE DRUG /CONTROL CONDITION	*none*
CONCOMITANT MEDICATION	Allowed – all concomitant medication is allowed<br>Patients who take cortisone on a regular basis will not be included in the study
EFFICACY ENDPOINTS	*not applicable*
TOLERABILITY / SAFETY ENDPOINTS	*not applicable*
PHARMACOKINETIC / PHARMACODYNAMIC ENDPOINTS	*Concentration of Triamcinolone e acetonide in perilymph and blood samples*
QUALITY OF LIFE / PHARMACOECONOMIC ENDPOINTS	*not applicable*
STATISTICAL METHODOLOGY	Primary objective:<br>The paired t-test will be used to compare the log-transformed perilymphatic and blood concentrations of triamcinolone, and a 95% confidence interval will be calculated for the geometric mean ratio.<br>Secondary objective:<br>Descriptive statistical methods will be used for the secondary (hypotheses generating) objective to compare the 4 groups of patients. Perilymphatic and blood concentrations of triamcinolone of the 4 groups will be graphically presented by boxplots, both on the original and on the log-transformed scale. Median (quartile) concentrations and means ± standard deviations of the log-transformed concentrations will be calculated.

3. LIST OF ABBREVATIONS

ITC	Intratympanic cortisone
i.v.	Intravenous
p.o.	Per os
SNHL	Sensorineural hearing loss
CI	Cochlear implant
AE	Adverse event
RWM	Round window membrane
SCC	Semicircular canal
CSF	Cerebrospinal fluid
SAE	Serious adverse event
SUSAR	Suspected unexpected serious adverse event

4. TABLE OF CONTENTS

CLINICAL STUDY PROTOCOL 1

1. SPONSOR, INVESTIGATOR, MONITOR AND SIGNATURES 3

2. PROTOCOL SYNOPSIS 5

3. LIST OF ABBREVATIONS 7

4. TABLE OF CONTENTS 8

5. BACKGROUND INFORMATION 12

BACKGROUND 12
STUDY RATIONALE 12

6. STUDY OBJECTIVES (HYPOTHESIS) 13

PRIMARY OBJECTIVE (HYPOTHESIS) 13
SECONDARY OBJECTIVES (HYPOTHESIS) 13

7. STUDY DESIGN 13

STUDY POPULATION 15
7.1.1 SUBJECT POPULATION 15
7.1.2 INCLUSION CRITERIA 15
7.1.3 EXCLUSION CRITERIA 15
7.1.4 FEMALES OF CHILDBEARING POTENTIAL 15
7.1.5 STUDY DURATION 15
7.1.6 WITHDRAWAL AND REPLACEMENT OF SUBJECTS 15
7.1.7 PREMATURE TERMINATION OF THE STUDY 16

8. METHODOLOGY 16

8.1 STUDY MEDICATION 18
8.1.1 DOSAGE AND ADMINISTRATION 19
8.1.2 STUDY-DRUG UP- AND DOWN TITRATION 19
8.1.3 STUDY-DRUG DELIVERY & DRUG STORAGE CONDITIONS 19
8.1.4 STUDY DRUG PACKAGING AND LABELING 19
8.1.5 IMP ADMINISTRATION & HANDLING 19

8.1.6 DRUG ACCOUNTABILITY 19
8.1.7 PROCEDURES TO ASSESS SUBJECTS COMPLIANCE 20
8.1.8 CONCOMITANT MEDICATION 20
8.2 RANDOMIZATION AND STRATIFICATION 20
8.3 BLINDING 20
8.4 BENEFIT AND RISK ASSESSMENT 20
8.5 STUDY PROCEDURES 20
8.5.1 GENERAL RULES FOR TRIAL PROCEDURES 20
8.5.2 SCREENING INVESTIGATION 21
8.5.3 END-OF-STUDY (EOS) EXAMINATION 21

9. SAFETY DEFINITIONS AND REPORTING REQUIREMENTS 21

ADVERSE EVENTS (AES) 21
9.1.1 SUMMARY OF KNOWN AND POTENTIAL RISKS OF THE STUDY DRUG 21
9.1.2 DEFINITION OF ADVERSE EVENTS 23
SERIOUS ADVERSE EVENTS (SAES) 24
9.1.3 HOSPITALIZATION – PROLONGATION OF EXISTING HOSPITALIZATION 25
9.1.4 SAES RELATED TO INVESTIGATIONAL DRUG 25
9.1.5 SUSPECTED UNEXPECTED SERIOUS ADVERSE REACTIONS (SUSARS) 25
9.1.6 PREGNANCY 25
SEVERITY OF ADVERSE EVENTS 26
RELATIONSHIP TO STUDY DRUG 27
REPORTING PROCEDURES 28
9.1.7 REPORTING PROCEDURES FOR SAES 28
9.1.8 REPORTING PROCEDURES FOR SUSAR 29
9.1.9 DEVELOPMENT SAFETY UPDATE REPORT 30

10. FOLLOW-UP 30

FOLLOW-UP OF STUDY PARTICIPANTS INCLUDING FOLLOW-UP OF ADVERSE EVENTS 30
TREATMENT AFTER END OF STUDY 30

11. STATISTICAL METHODOLOGY AND ANALYSIS 30

ANALYSIS SETS 31
SAMPLE SIZE CONSIDERATIONS 31
RELEVANT PROTOCOL DEVIATIONS 31
ENDPOINTS ANALYSIS 31
11.1.1 PRIMARY ENDPOINT ANALYSIS 31
11.1.2 SECONDARY ENDPOINT ANALYSIS 32
INTERIM ANALYSIS 32

12. DOCUMENTATION AND DATA MANAGEMENT 32

DOCUMENTATION OF STUDY RESULTS **32**
12.1.1 CASE REPORT FORM (CRF) 32
12.1.2 DATA COLLECTION 33
SAFEKEEPING **33**
QUALITY CONTROL AND QUALITY ASSURANCE **33**
12.1.3 PERIODIC MONITORING 33
12.1.4 AUDIT AND INSPECTIONS 34
REPORTING AND PUBLICATION **34**
12.1.5 PUBLICATION OF STUDY RESULTS 34

13. ETHICAL AND LEGAL ASPECTS **34**

INFORMED CONSENT OF SUBJECTS **34**
ACKNOWLEDGEMENT / APPROVAL OF THE STUDY **35**
13.1.1 CHANGES IN THE CONDUCT OF THE STUDY 35
INSURANCE **36**
CONFIDENTIALITY **36**
ETHICS AND GOOD CLINICAL PRACTICE (GCP) **37**

14. REFERENCES **38**

TABLE 1. VISIT AND ASSESSMENT SCHEDULE

		Screening	Treatment		Sampling	Follow Up		
	Duration		1 day		1 day	1 week		
Visits	Number	1	2	3	4	5	6	7
	Name	Screening	Randomization	Application	Sampling	Control 1	Control 2	Control 3
	Time		day 0	day 0 (gr. 1+2)<br>day 1 (gr. 3+4)	day 1	day 2	day 3	1 week<br>+/- 2d
Informed Consent		x						
Inclusion / Exclusion Criteria		x	x					
Medical History		x	x					
Concomitant medication		x	x					
ENT Status		x	x					
Ear microscopy		x	x	x		x		x
Body weight and height			x					
Puretonaudiogramm		x	x			x		
Intratympanic Triamcinolone Application				x				
Perilymph Sample					x			
Blood Sample					x			
Adverse Events				x	x	x	x	x

5. BACKGROUND INFORMATION

Background

Over the past few years intratympanic cortisone application has been established additionally to intravenous cortisone application in the therapy of sudden sensorineural hearing loss (SNHL), toxic acute middle ear infections and preoperatively before operations of the inner ear (Cochlear implantation). Intratympanic application leads to a higher steroid concentration in the cochlear perilymph than i.v. administration does [1, 2]. Steroids are known for having several otoprotective qualities.
When performing a cochlear implantation corticosteroid therapy can protect the residual hearing
[3]. Hair cells of the inner ear are protected by steroids, when a trauma is induced [4].
Studies have shown that glucocorticoids reduce the impedance needed by the cochlear implant (CI). By the reduction of impedances needed a battery sparing function of the CI is made possible [5].
Inflammation, which leads to fibrosis in the cochlea and around the electrode, can also be reduced by steroids [5].
The intratympanic application of steroids is used as salvage therapy in patients with sudden SNHL [6] or as primary treatment option for patients who cannot be treated systemically.
When applying steroids intratympanically the effect seems to be in the applied area and systemic side effects can be avoided.
At our department a similar study was conducted on guinea pigs. The method of detection of Triamcinolone acetonide in the perilymph has already been established [7].

Study rationale

Two studies have been published so far analyzing perilymph concentrations of prednisolone after intravenous administration [8, 9]. Both studies could show that higher prednisolone doses result in higher perilymphatic concentrations. Two further studies examined blood and perilymphatic levels after intratympanic administration – one using methylprednisolone and the other one using dexamethasone [10] [2]. Both studies could show that intratympanic application leads to a very small dissemination of the medication to the blood circulation and to high perilymphatic concentrations compared to intravenous application.
When administering liquids to the middle ear they are rapidly drained via the Eustachian tube and the effect is lost. None of those studies investigated a crystalline/depot compound.

In Austria Triamcinolone acetonide (Volon® A) is the steroid of choice for intratympanic application. As for other glucocorticoids applied intratympanically (IT) the application of Volon® A through the tympanic membrane corresponds an "Off-Label-Use". The IT application has already been integrated in treatment guidelines of sudden SNHL and therefor is carried out at our department as part of routine procedures.
Because of the crystalline compound of Volon® A a depot effect is presumed. The otoprotective value of Triamcinolone acetonide could already be shown in studies [11, 12]. Up to now, no study has shown how much Triamcinolone acetonide is absorbed by the perilymph. Furthermore, the presumed depot effect is only proven for other uses. If Triamcinolone acetonide leads to a longer lasting and higher perilymph level has not been proven so far. To what extent Triamcinolone acetonide is disseminated to the blood circulation after intratympanic application has not been investigated yet.

6. STUDY OBJECTIVES (HYPOTHESIS)

Primary Objective (Hypothesis)

Demonstrate concentration of Triamcinolone in cochlear perilymph in comparison to concentrations in the blood.

Secondary Objectives (Hypothesis)

Assess the stability of triamcinolone acetonide levels in the cochlear perilymph.
Assess the difference of triamcinolone acetonide levels in the cochlear perilymph and blood after administration of different doses of Triamcinolone acetonide.

7. STUDY DESIGN

At the ENT department of the university hospital Vienna (AKH Wien) patients are treated with intratympanic triamcinolone acetonide before cochlea implantation to reduce inflammation and in some cases to protect residual hearing. Triamcinolone acetonide levels in cochlear perilymph will be evaluated in an open prospective clinical study. Patients scheduled for cochlear implant surgery between 18 and 90 years will be included. Patients who are treated with steroids preoperatively will be excluded from the study. Patients will be randomized after inclusion to one of four groups. The randomization is carried out to generate hypothesis for the needed dose and best time of application in the future. Triamcinolone acetonide will then be applied 20-24h before surgery or at the beginning of the surgery, depending on randomization (see below). About 20 µl of perilymph will be sampled simultaneously to a

blood sample during cochlear implant surgery. The first twenty patients will be randomized to Triamcinolone acetonide 40mg to allow for a first analysis of the samples. Patients 21 to forty will be randomized to Triamcinolone acetonide 10mg.
The probes will be stored at -80°C. Triamcinolone acetonide levels of the blood and perilymph will be determined by the pharmaceutical laboratory (Department of Pharmaceutical Technology and Biopharmaceutics, University of Vienna).
The patients will be randomized to 4 groups. Group 1 - Volon A® 10mg administration 20 - 24 hours before sampling. Group 2 – Volon® A 40mg administration 20 - 24 hours before sampling. Group 3 Volon® A 10mg – administration 1 to 2 hours before sampling. Group 4 – Volon A® 40mg administration 1 to 2 hours before sampling.
The time interval of application (1 to 2 hours and 20 to 24 hours before sampling) are a result of varying time of surgery depending on surgeons and patient anatomy as well as day to day clinical organization. Patients can withdraw consent at any time of the study.
The active phase of each patient will be between 6 and 9 days depending on time of follow-up visit.

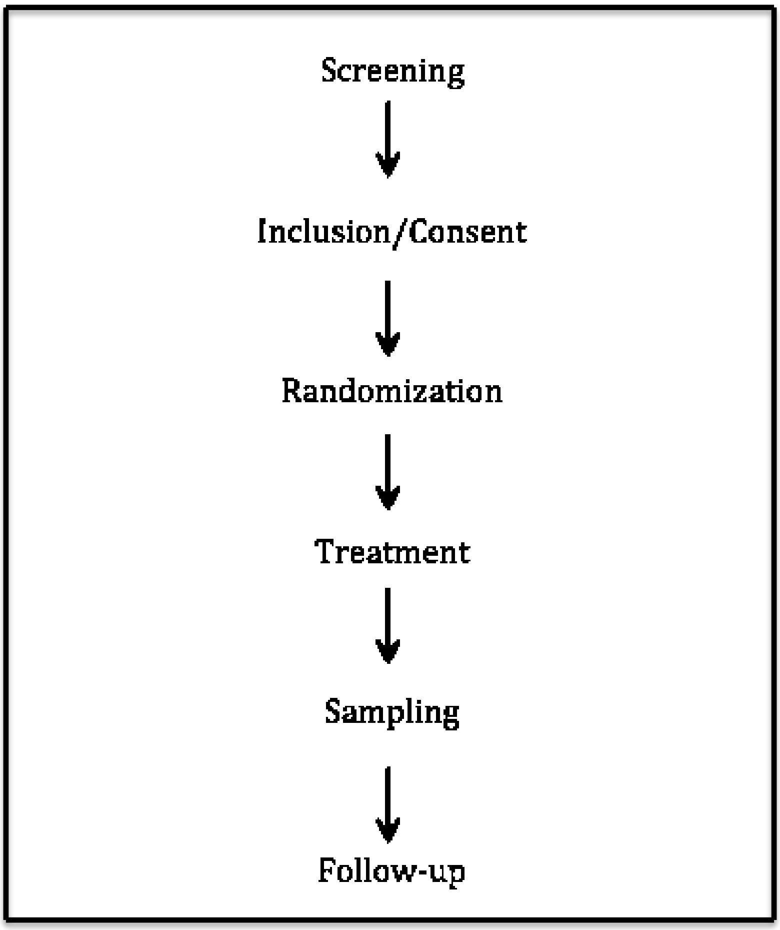

Study population

7.1.1 Subject population

Patients, between 18 and 90 years, will be included in the study, who are scheduled to receiving a cochlear implant and who are willing to participate in the study.

7.1.2 Inclusion criteria

- Patients between 18 and 90 years receiving a cochlear implant and who are willing to participate in the study.

7.1.3 Exclusion criteria

- Patients younger than 18 years
- Patients who receive cortisone regularly or receive cortisone i.v. or p.o. preoperatively
- Patients with contraindications against the administration of Volon® A

7.1.4 Females of childbearing potential

- Females of childbearing potential will be included in the study, if birth control is carried out (hormonal contraception, intrauterine or barrier contraception) or a pregnancy can be ruled out completely.

7.1.5 Study duration

- Patients will be recruited over a time period of 2 years. The active phase of each patient will last for up to 9 days.

7.1.6 Withdrawal and replacement of subjects

Criteria for withdrawal

Subjects may prematurely discontinue from the study at any time.

Subjects must be withdrawn under the following circumstances:

- at their own request
- if the Investigator feels it would not be in the best interest of the subject to continue

- if the subject violates conditions laid out in the consent form or disregards instructions by the study personal

In all cases, the reason why subjects are withdrawn must be recorded in detail in the CRF and in the subject's medical records. Should the study be discontinued prematurely, all study materials (completed, partially completed and empty CRFs) will be retained.

Follow-up of patients withdrawn from the study

Patients routinely receive intratympanic Triamcinolone acetonide. The study only consists of the calculation of Triamcinolone acetonide concentration in the perilymph and blood. Patients will be asked for adverse events at routine post-operative follow-up visits. No further follow up visits are necessary or planned within this study.

7.1.7 Premature termination of the study

The sponsor has the right to close this study at any time. The IEC and the competent regulatory authority must be informed within 15 days of early termination.
The trial or single dose steps will be terminated prematurely in the following cases:

- If adverse events occur which are so serious that the risk-benefit ratio is not acceptable.
- If the number of dropouts is so high that proper completion of the trial cannot realistically be expected.

8. METHODOLOGY

At the department of the Medical University of Vienna patients routinely receive intratympanic application of triamcinolone acetonide.
1 mL suspension containing 10mg (Groups 1 and 3) or 40mg (Groups 2 and 4) of triamcinolone acetonide is applied intratympanically via a 25G (0.50x90mm, 3.50 IN) needle after local anesthesia with xylocaine spray (10mg/Puff).
During the cochlear implant surgery, the cochlea is entered mostly via round window approach. Alternatively, if the round window cannot be identified, a cochleostomy is performed. After entering the cochlea, about 20µL of perilymph are sampled using a sterile disposable aspirator with a diameter of 0.4mm. A sterile insulin syringe is used to aspirate the perilymph. The perilymph sampling method has been performed similarly in other studies [2, 10].

Simultaneously a blood sample of 4ml is drawn in a heparin blood tube with a standard butterfly needle.
The sample of perilymph is stored in an eppendorf vial (volume 0,2ml) and frozen to approx. -80°.
The blood sample is centrifuged at 3000g, 20°C for 3 minutes at. The plasma is removed and stored in eppendorf vials (volume 2ml) and also frozen to approx.-80°. Both vials will be labeled with the patient number. Patients will be numbered with a randomly assigned three-digit number.

Quantification of Triamcinolone acetonide

Samples will be diluted with mobile phase consisting of acetonitrile/2 mM aqueous ammonium acetate (60:40) adjusted to pH 3.2 with formic acid [13] and stored at +4 ° C until analysis by high-performance liquid chromatography/mass spectrometry, comprising an Ultimate RSLC 3000 series System (Thermo Fisher Scientific, Vienna, Austria) and an API 4000 Triple Quadrupole Mass Spectrometer (AB Sciex instruments, Vienna, Austria) equipped with an electro- spray ionization ion source and controlled by the Analyst 1.5 soft- ware (Dionex, Vienna, Austria). The chromatographic separation of samples will be conducted by isocratic elution using an Acclaim® 120 C_{18} reversed-phase LC column (2.1 × 150 mm, 3 μm; Thermo Fisher Scientific, Vienna, Austria) at 25 ° C. The run time will be 15 min at a flow rate of 0.5 ml/min, and the retention time of TAAc is about 1.28 min. Released TAAc will be selectively detected and quantified by tandem mass spectrometry fragmentation giving a quasimolecular ion at m/z 435 [M-H]+. Each sample series will be monitored by quality control samples containing certain amounts of TAAc within the range of the calibration graph.

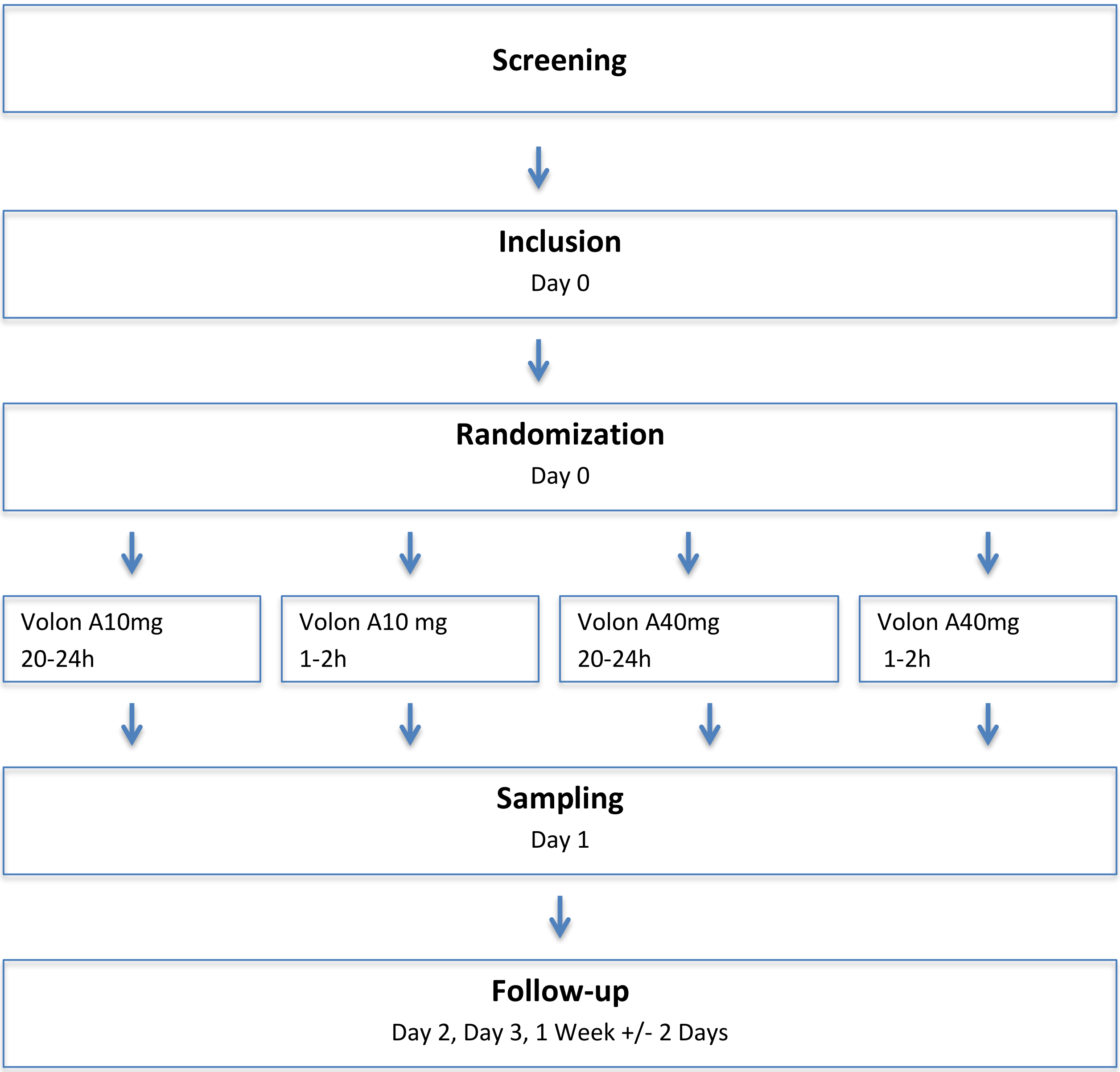

8.1 Study medication

Active agent and characteristics: Triamcinolone acetonide

Trade name of the agent: Volon® A crystal suspension

Manufacturer: Mibe GmbH Arzneimittel

Drug supply: Dermapharm GmbH

Storage Instructions: Freezing is not allowed. Volon® A should be stored in the box to protect in from light.

Route of administration: Intratympanic

Information can be found in the investigator's brochure.

8.1.1 Dosage and administration

Dose: 1mL of Volon® A 10mg or 40mg

Route of administration: intratympanic

Duration: single administration

8.1.2 Study-drug up- and down titration

Not feasible

8.1.3 Study-drug delivery & drug storage conditions

Triamcinolone acetonide is delivered at room temperature. The content has to be stored shielded from light, in the boxing. Triamcinolone acetonide should not be frozen.

It will be stored in the packaging at room temperature at the ENT department (ward 15J or 15I).

8.1.4 Study drug packaging and labeling

Since Triamcinolone acetonide is a registered drug it will be stored in the original package.

8.1.5 IMP administration & handling

1 mL Triamcinolone acetonide is applied intratympanically via a 25GA (0.50x90mm, 3.50 IN) needle after local anesthesia with Xylocaine spray (10mg/Puff).

Triamcinolone acetonide should be inspected before use. If agglomerations occur the drug should not be administered.

8.1.6 Drug Accountability

The batch number will be documented on the Case Report Form. Triamcinolone acetonide will be administered at a single time point by a medical doctor intratympanically.

8.1.7 Procedures to assess subject's compliance

Not applicable

8.1.8 Concomitant medication

Allowed: All concomitant medication is allowed. Patients who have to take cortisone preoperatively will not be included in the study.

8.2 Randomization and stratification

For Randomization https://www.meduniwien.ac.at/randomizer will be used. The first twenty patients will be randomized to the 40mg groups. The second half will be randomized to the 10mg groups. On day 0 participants will be randomized to one of 2 groups and the intratympanic application of triamcinolone will be administered accordingly on day 0 or day 1. Block randomization will be used in groups of 8. The method of permuted blocks randomization will be used with an equally weighted allocation to the 4 treatment groups (resulting in 10 patients per group).

8.3 Blinding

No blinding is required in this study.

8.4 Benefit and risk assessment

This study consists of a perilymph and a blood sample during cochlear implantation. Patients included in the study are deaf or profoundly hearing impaired. As previously shown in other studies sampling of the perilymph is a safe method.
This study can give us information on the absorption of Triamcinolone acetonide by perilymph as well as information on the stability of Triamcinolone acetonide after absorption of the perilymph. Since Triamcinolone acetonide is used in the treatment of sudden SNHL and preoperatively, this study can deliver necessary information on the best timing of drug application and the necessary dose.
Additionally, a blood sample is taken, which can lead to known risks such as infections and hematoma.

8.5 Study procedures

8.5.1 General rules for trial procedures

- All study measures like blood sampling and measurements have to be documented with date (dd:mm:yyyy).
- In case several study procedures are scheduled at the same time point, there is no specific sequence that should be followed.

- The dates of all procedures should be according to the protocol. The time margins mentioned in the study flow chart are admissible. If for any reason, a study procedure is not performed within scheduled margins a protocol deviation should be noted, and the procedure should be performed as soon as possible or as adequate.
- If it is necessary for organizational reasons, it is admissible to perform procedures, which are scheduled for one visit at two different time points. Allowed time margins should thereby not be exceeded.

8.5.2 Screening investigation

Patients scheduled for cochlear implantation will be asked to participate in the study. Inclusion and exclusion criteria will be evaluated at the end outpatient’s department.
If patients fulfill inclusion and exclusion criteria and consent to the study is given a reevaluation on day 0 will be carried out. If the second evaluation has a positive outcome patients will then be included in the study on day 0.

8.5.3 End-of-study (EOS) examination

One week after surgery patients routinely are scheduled for wound examination and stitch removal as well as scheduling of cochlear implant fitting. During this examination adverse events will be evaluated. Additionally, ear microscopy is carried out.

9. SAFETY DEFINITIONS AND REPORTING REQUIREMENTS

Adverse events (AEs)

9.1.1 Summary of known and potential risks of the study drug

Known side effects:

Cardiovascular

Arrhythmia, heart failure

Musculoskeletal

Aseptic necrosis of femoral and humeral heads, loss of muscle mass, muscle weakness, osteoporosis, pathologic fracture of long bones, tendon rupture, and vertebral compression

fractures, bone mineral density loss and osteoporosis, steroid myopathy, vasculitis

Gastrointestinal

Peptic ulcer with potential perforation and hemorrhage, perforation of small and large bowel, pancreatitis, abdominal distention and ulcerative esophagitis

Dermatologic

Impaired wound healing, thin, fragile skin, petechiae and ecchymoses, facial erythema, increased sweating, and suppressed reactions to skin tests, purpura, striae rubrae, hyperpigmentation, steroidakne, allergic dermatitis

Nervous system

Convulsions, increased intracranial pressure with papilledema, vertigo and headache, pseudotumor cerebri, manifestation of a latent epilepsie, sleeping disorders, neuritis, paresthesia

Endocrine

Menstrual irregularities, postmenopausal vaginal bleeding, hirsutism, suppression of growth in children, manifestations of latent diabetes, increased requirements for insulin or oral hypoglycemic agents in diabetics, decreased carbohydrate tolerance, and secondary adrenocortical and pituitary unresponsiveness, impotence, pseudo-cushing syndrome, weight gain, negative protein and calcium balance, increased appetite

Ocular

Cataract, glaucoma, increased intraocular pressure, glaucoma and exophthalmos, cornea perforation

Hypersensitivity

Anaphylactoid reactions, anaphylaxis, and angioedema

Vascular diseases

Necrotizing angiitis, higher risk of artheriosis and thrombosis

Electrolyte dysbalance

Natrium retention, water retention, higher potassium excretion, hypokalemic alkalosis,

hypertension, hyperglycemia, glukosuria

Other

Anaphylactic reaction, feeling of heat

Medications with potential interactions:

Cardiac glycosides
ACE inhibitors
Chloroquine, Hydrochloroquine, Mefloquine
Aspirin
NSAIDs
Oral Anticoagulants
Bupropion
Methotrexate

9.1.2 Definition of adverse events

An AE is any untoward adverse change from the subject's baseline condition, i.e., any unfavorable and unintended sign including an abnormal laboratory finding, symptom or disease which is considered to be clinically relevant by the physician that occurs during the course of the study, whether or not considered related to the study drug.
Adverse events include:

- Exacerbation of a pre-existing disease.
- Increase in frequency or intensity of a pre-existing episodic disease or medical condition.
- Disease or medical condition detected or diagnosed after study drug administration even though it may have been present prior to the start of the study.
- Continuous persistent disease or symptoms present at baseline that worsen following the start of the study.
- Lack of efficacy in the acute treatment of a life-threatening disease.
- Events considered by the Investigator to be related to study-mandated procedures.
- Abnormal assessments, e.g., ECG and physical examination findings, must be reported as AEs if they represent a clinically significant finding that was not present at baseline or worsened during the course of the study.

- Laboratory test abnormalities must be reported as AEs if they represent a clinically significant finding, symptomatic or not, which was not present at baseline or worsened during the course of the study or led to dose reduction, interruption or permanent discontinuation of study drug.

Adverse events do not include:

- Pre-planned interventions or occurrence of endpoints specified in the study protocol are not considered AE´s, if not defined otherwise (eg.as a result of overdose)
- Medical or surgical procedure, e.g., surgery, endoscopy, tooth extraction, transfusion. However, the event leading to the procedure is an AE. If this event is serious, the procedure must be described in the SAE narrative.
- Pre-existing disease or medical condition that does not worsen.
- Situations in which an adverse change did not occur, e.g., hospitalizations for cosmetic elective surgery or for social and/or convenience reasons.
- Overdose of either study drug or concomitant medication without any signs or symptoms. However, overdose must be mentioned in the Study Drug Log.

Serious Adverse Events (SAEs)

A Serious Adverse Event (SAE) is defined by the International Conference on Harmonization (ICH) guidelines and GCP guidelines as any AE fulfilling at least one of the following criteria:

- Results in deaths.
- Life-threatening – defined as an event in which the subject was, in the judgment of the Investigator, at risk of death at the time of the event;
- Requiring subject's hospitalization or prolongation of existing hospitalization
- Resulting in persistent or significant disability or incapacity (i.e., a substantial disruption of a person’s ability to conduct normal life functions).
- Congenital anomaly or birth defect.
- Optional: Is medically significant or requires intervention to prevent at least one of the outcomes listed above

Life threatening refers to an event in which the subject was at risk of death at the time of the event. It does not refer to an event that hypothetically might have caused death if it were more severe.

Important medical events that may not immediately result in death, be life-threatening, or require hospitalization may be considered as SAEs (optional!) when, based upon appropriate

medical judgment, they may jeopardize the subject and may require medical or surgical intervention to prevent one of the outcomes listed in the definitions above. This means an individual case decision.

9.1.3 Hospitalization – Prolongation of existing hospitalization

Hospitalization is defined as an overnight stay in a hospital unit and/or emergency room.
An additional overnight stay defines a prolongation of existing hospitalization.
The following is not considered an SAE and should be reported as an AE only:

- Treatment on an emergency or outsubject basis for an event not fulfilling the definition of seriousness given above and not resulting in hospitalization.

The following reasons for hospitalizations are not considered AEs, and therefore not SAEs:

- Hospitalizations for cosmetic elective surgery, social and/or convenience reasons.
- Elective treatment of a pre-existing disease or medical condition that did not worsen, e.g., hospitalization for chemotherapy for cancer, elective hip replacement for arthritis.

9.1.4 SAEs related to investigational drug

Such SAEs are defined as SAEs that appear to have a reasonable possibility of causal relationship.

9.1.5 Suspected unexpected serious adverse reactions (SUSARs)

SUSARs are all serious adverse reactions with **suspected** causal relationship to the study drug that is **unexpected** (not previously described in the Summary of Product Characteristics or Investigator’s brochure) and serious. SUSARs will be reported within 24hours.

9.1.6 Pregnancy

Any pregnancy that occurs during study participation must be reported to the Investigator/sponsor. To ensure subject safety, each pregnancy must be reported to the Sponsor immediately. The pregnancy must be followed up to determine outcome (including premature termination) and status of mother and child. Pregnancy complications and

elective terminations for medical reasons must be reported as an AE or SAE. Spontaneous abortions must be reported as an SAE.
Any SAE occurring in association with a pregnancy brought to the Investigator's attention after the subject has completed the study and considered by the Investigator as possibly related to the investigational product, must be promptly reported to the Investigator/sponsor.
In addition, the Investigator must attempt to collect pregnancy information on any female partners of male study subjects who become pregnant while the subject is enrolled in the study. Pregnancy information must be reported to the Investigator/sponsor as described above.

Severity of adverse events

The severity of clinical AEs is graded on a three-point scale: mild, moderate, severe, and reported on specific AE pages of the CRF.
If the severity of an AE worsens during study drug administration, only the worst intensity should be reported on the AE page. If the AE lessens in intensity, no change in the severity is required.
If an AE occurs during a washout or placebo run-in phase and afterwards worsens during the treatment phase, a new AE page must be filled in with the intensity observed during study drug administration.

Mild
Event may be noticeable to subject; does not influence daily activities; the AE resolves spontaneously or may require minimal therapeutic intervention;

Moderate
Event may make subject uncomfortable; performance of daily activities may be influenced; intervention may be needed; the AE produces no sequelae.

Severe
Event may cause noticeable discomfort; usually interferes with daily activities; subject may not be able to continue in the study; the AE produces sequelae, which require prolonged therapeutic intervention.

A mild, moderate or severe AE may or may not be serious. These terms are used to describe the intensity of a specific event (as in mild, moderate, or severe myocardial infarction). However, a severe event may be of relatively minor medical significance (such as severe headache) and is not necessarily serious. For example, nausea lasting several hours may be rated as severe, but may not be clinically serious. Fever of 39°C that is not considered severe may become serious if it prolongs hospital discharge by a day. Seriousness rather than severity serves as a guide for defining regulatory reporting obligations.

Relationship to study drug

For all AEs, the Investigator will assess the causal relationship between the study drug and the AE using his/her clinical expertise and judgment according to the following algorithm that best fits the circumstances of the AE:

Not related

- May or may not follow a temporal sequence from administration of the study product
- Is biologically implausible and does not follow known response pattern to the suspect study drug (if response pattern is previously known).
- Can be explained by the known characteristics of the subject’s clinical state or other modes of therapy administered to the subject.

Unlikely

- There is a reasonable temporal relation between the AE and the intake of the study medication, but there is a plausible other explanation for the occurrence of the AE.

Possibly

- The AE has a reasonable temporal relationship with drug administration.
- The AE may equally be explained by the study subject’s Clinically state, environmental or toxic factors, or concomitant therapy administered to the study subject.
- The relationship between study drug and AE may also be pharmacologically or clinically plausible.

Probably

- There is a reasonable temporal relation between the AE and the intake of the study medication, and plausible reasons point to a causal relation with the study medication.

Related

- Reasonable temporal relation between the AE and the intake of the study medication and
- there is no other explanation for the AE and
- subsidence or disappearance of the AE on withdrawal of the study medication and
- recurrence of the symptoms on restart at previous dose (only applies for re-institution of mediation).

Not assessable

- The causal relationship between the study drug and the AE cannot be judged.

Reporting procedures

A special section is designated to adverse events in the case report form. The following details must thereby be entered:

- Type of adverse event
- Start (date and time)
- End (date and time)
- Severity (mild, moderate, severe)
- Serious (no / yes)
- Unexpected (no / yes)
- Outcome (resolved, resolving, not resolved, resolved with sequelae, unknown, fatal)
- Relation to study drug (Related/ Probably/ Possibly/ Unlikely/ Not related/ Not assessable)

Adverse events are to be documented in the case report form in accordance with the above-mentioned criteria.

9.1.7 Reporting procedures for SAEs

In case of a serious Adverse event, the Investigator has to use all supportive measures for best patient treatment. A written report is also to be prepared and should at least contain the following:

- Patient number
- Patient: sex
- The suspected investigational medical product (IMP)
- The adverse event assessed as serious
- Short description of the event and outcome

If applicable, the initial report should be followed by the Follow up report, indicating the outcome of the SAE.

9.1.8 Reporting procedures for SUSAR

It must be remembered that the regulatory authorities, and the Institutional Review Board / Independent Ethics Committee (IRB / IEC) must be informed about all SUSAR. Such reports shall be made by the sponsor and should content at least the following details:

- Patient number (study code/screening number)
- Patient: age in years, sex
- Name of Investigator and investigating site
- Period of administration
- The suspected investigational medical product (IMP)
- The adverse event assessed as serious and unexpected, and for which there is a **suspected** causal relationship to the IMP
- Concomitant disease and medication
- Short description of the event:
    - Description
    - Onset and if applicable, end
    - Therapeutic intervention
    - Causal relationship
    - Seriousness criteria or reportable reason

Electronic reporting should be the expected method for reporting of SUSARs to the competent authority. In that case, the format and content as defined by the regulatory requirements should be adhered to. The latest version of MedDRA should be applied. Lower level terms (LLT) should be used.

9.1.9 Development Safety Update Report

A Development Safety Update Report (DSUR) will be provided by the Sponsor annually. This report will also be presented annually to the Independent Ethics (IEC) and to the competent authorities by the sponsor.

10. FOLLOW-UP

Follow-up of study participants including follow-up of adverse events

Patients will be at the hospital at least two days after surgery (surgery = day 1). During this time patients will be asked for any adverse events on a daily basis. Additionally, patients will be followed up one week after surgery for wound inspection and stitch removal. At this follow-up visit patients will be asked for adverse events as well.

Treatment after end of study

There will be no treatment after end of study.

11. STATISTICAL METHODOLOGY AND ANALYSIS

Statistical methodology

Primary objective:
The paired t-test will be used to compare the log-transformed perilymphatic and blood concentrations of Triamcinolone acetonide, and a 95% confidence interval will be calculated for the geometric mean ratio.
Secondary objective:
Descriptive statistical methods will be used for the secondary (hypotheses generating) objective to compare the 4 groups of patients. Perilymphatic and blood concentrations of

Triamcinolone acetonide of the 4 groups will be graphically presented by boxplots, both on the original and on the log-transformed scale. Median (quartile) concentrations and means ± standard deviations of the log-transformed concentrations will be calculated.

Data handling

Case report forms will be copied and stored separately. Data will be double-checked and saved in an excel sheet on a computer to which only study personnel has access to. Patients will be randomly assigned a three-digit number and the further data analysis will be carried out anonymously.

Analysis sets

Intention to treat set

This analysis set includes subjects who were randomized (and received at least one dose study drug).

Sample size considerations

The sample size calculation is based on the paired t-test and a two-sided significance level of 5%. Perilymphatic and blood concentrations of 12 patients are known from the literature (Bird et al, 2011) with geometric mean values of 1.26 and 0.001, respectively, and the standard deviation of the paired differences of the log10-transformed values was calculated as 0.81. To detect an effect size of 0.5 standard deviations, which is a difference of 0.4 on the log-scale (a difference in the geometric means of 1.26 to 0.5) with a statistical power of 86%, a total number of 40 patients have to be included in the study (nQuery Advisore 7.0).

Relevant protocol deviations

All protocol deviations will be listed in the study report and noted on case report forms.

Endpoints analysis

11.1.1 Primary endpoint analysis

Perilymphatic and blood concentration of Triamcinolone acetonide

<u>Primary objective:</u>

The paired t-test will be used to compare the log-transformed perilymphatic and blood concentrations of Triamcinolone acetonide, and a 95% confidence interval will be calculated for the geometric mean ratio.

11.1.2 Secondary endpoint analysis

Secondary objective:

Descriptive statistical methods will be used for the secondary (hypotheses generating) objective to compare the 4 groups of patients. Perilymphatic and blood concentrations of Triamcinolone acetonide of the 4 groups will be graphically presented by boxplots, both on the original and on the log-transformed scale. Median (quartile) concentrations and means ± standard deviations of the log-transformed concentrations will be calculated.

Interim analysis

Not applicable

Criteria for the termination of the trial

See 7.1.7

12. DOCUMENTATION AND DATA MANAGEMENT

Documentation of study results

A subject screening and identification log will be completed for all screened subjects with the reasons for exclusion.

12.1.1 Case report form (CRF)

For each subject enrolled, regardless of study drug initiation, a CRF must be completed and signed by the Investigator or a designated sub-Investigator. This also applies to those subjects who fail to complete the study. If a subject withdraws from the study, the reason must be noted on the CRF. Case report forms are to be completed on an ongoing basis. CRF entries and corrections will only be performed by study site staff, authorized by the Investigator.

In a paper based CRF all forms should be completed and must be legible. Entry errors have to be corrected according the ICH-GCP Guidelines.

The entries will be checked by trained personnel (Monitor) and any errors or inconsistencies will be corrected immediately.
The monitor will collect original completed and signed CRFs at the end of the study. A copy of the completed and signed CRFs will remain on site, as will the original data.
Original CRFs will be passed to study personnel (Data Manager). Paper based CRFs will be used.

12.1.2 Data Collection

Data collected at all visits are entered into an interactive form. The CRFs will be source documents verified following guidelines established before study onset as detailed in the Monitoring Plan. Maintenance of the study database will be performed by study personnel.

Safekeeping

The Investigator will maintain adequate and accurate records to enable the conduct of the study to be fully documented and the study data to be subsequently verified (according to ICH-GCP “essential documents”). These documents will be classified into two different categories: Investigator's study site file (ISF) with all essential documents regarding the study conduct, and subject clinical source documents.
The Investigator's file will contain all essential documents listed in ICH-GCP Guidelines section 8.
Subject clinical source documents include all patient hospital clinical records in original version, such as original laboratory reports, ECG, X-ray prints and other reports.

These two categories of documents must be kept on file by the Investigator for as long as needed to comply with the regulatory requirements.

Quality Control and Quality Assurance

12.1.3 Periodic Monitoring

The designated monitor will contact and visit the Investigator on a regularly basis and will be allowed to have direct access to all source documents needed to verify the entries in the

CRFs and other protocol-related documents provided that subject confidentiality is maintained in agreement with local regulations. It will be the monitor's responsibility to inspect the CRFs at regular intervals according to the monitoring plan throughout the study, to verify the adherence to the protocol and the completeness, consistency and accuracy of the data being entered on them.
A qualified staff member of the ENT department will perform monitoring. An initiation visit, a control visits and a close out visit will be carried out. The monitor will randomly check source data, all patient consent forms, storage of study medication and accordance to study protocol.

12.1.4 Audit and Inspections

Upon request, the Investigator will make all study-related source data and records available to a qualified quality assurance auditor mandated by the sponsor or to competent authority inspectors. The main purposes of an audit or inspection are to confirm that the rights and welfare of the subjects have been adequately protected, and that all data relevant for assessment of safety and efficacy of the investigational product have appropriately been reported to the sponsor.

Reporting and Publication

12.1.5 Publication of study results

The findings of this study will be published by the sponsor (Investigators) in a scientific journal and presented at scientific meetings. The manuscript will be circulated to all co-Investigators before submission. Confidentiality of subjects in reports/publications will be guaranteed.

13. ETHICAL AND LEGAL ASPECTS

Informed consent of subjects

Following comprehensive instruction regarding the nature, significance, impact and risks of this clinical trial, the patient must give written consent to participation in the study.

During the instruction the trial participants are to be made aware of the fact that they can withdraw their consent – without giving reasons – at any time without their further medical care being influenced in any way.
In addition to the comprehensive instructions given to the trial participants by the Investigator, the trial participants also receive a written patient information sheet in comprehensible language, explaining the nature and purpose of the study and its progress. The patients must agree to the possibility of study-related data being passed on to relevant authorities.
The patients must be informed in detail of their obligations in relation to the trial participants insurance in order not to jeopardize insurance cover.

Acknowledgement / approval of the study

The Investigator (or a designated CRO) will submit this protocol and any related document provided to the subject (such as subject information used to obtain informed consent) to an Ethics Committee (EC) or Institutional Review Board (IRB). Approval from the committee must be obtained before starting the study.
The clinical trial shall be performed in full compliance with the legal regulations according to the Drug Law (AMG - Arzneimittelgesetz) of the Republic of Austria.
An application must also be submitted to the Austrian Competent Authorities (Bundesamt für Sicherheit im Gesundheitswesen (BASG) represented by the Agency for Health and Food Safety (AGES Medizinmarktaufsicht) and registered to the European Clinical Trial Database (EudraCT) using the required forms. The timelines for (silent) approval set by national law must be followed before starting the study.

13.1.1 Changes in the Conduct of the Study

Protocol amendments
Proposed amendments must be submitted to the appropriate CA and ECs. Substantial amendments may be implemented only after CA/EC approval has been obtained.
Amendments that are intended to eliminate an apparent immediate hazard to subjects may be implemented prior to receiving CA/EC approval. However, in this case, approval must be obtained as soon as possible after implementation.

Study Termination

If the sponsor or the Investigator decides to terminate the study before the planned completion, they will notify each other in writing stating the reasons of early termination. Both the sponsor and the investigator will ensure the protection of the subject's wellbeing. The sponsor will notify the regulatory authority as well as the ethics committee about the premature termination. Documentation will be filed in the Trial Master File as well as in the Investigator Site File.

Clinical Study Report (CSR)

Within one year after the final completion of the study, a full CSR will be prepared by the sponsor and submitted to the EC and the competent authority.
The Investigator will be asked to review and sign the final study report.

Insurance

During their participation in the clinical trial the patients will be insured as defined by legal requirements. The Investigator of the clinical trial will receive a copy of the insurance conditions of the 'patients' insurance'. The sponsor is providing insurance in order to indemnify (legal and financial coverage) the Investigator/center against claims arising from the study, except for claims that arise from malpractice and/or negligence. The compensation of the subject in the event of study-related injuries will comply with the applicable regulations. Details on the existing patient's insurance are given in the patient information sheet.
Patients will be insured according to the general agreement of the Medical university of Vienna:

Zürich Versicherungs AG
Schwarzenbergplatz 15
1010 Wien
Tel.: 0043 (01) 50125-0

Confidentiality

The information contained in this document, especially unpublished data, is the property of the sponsor of this study. It is therefore provided to you in confidence as an Investigator, potential Investigator, or consultant, for review by you, your staff, and an Ethics Committee or Institutional Review Board. It is understood that this information will not be disclosed to others without written authorization from the Sponsor of this study (Prof. W. Gstöttner).

Ethics and Good Clinical Practice (GCP)

The Investigator will ensure that this study is conducted in full conformance with the principles of the "Declaration of Helsinki" (as amended at the 64th WMA General Assembly, Fortaleza, Brazil, 2013) and with the laws and regulations of the country in which the clinical research is conducted.

The Investigator of the clinical trial shall guarantee that only appropriately trained personnel will be involved in the study. All studies must follow the ICH GCP Guidelines and the regulatory requirements.

Therefore, this study follows the EU Directive embedded in the Austrian drug act.

14. REFERENCES

1. Hobson, C.E., T.H. Alexander, and J.P. Harris, *Primary treatment of idiopathic sudden sensorineural hearing loss with intratympanic dexamethasone.* Curr Opin Otolaryngol Head Neck Surg, 2016. **24**(5): p. 407-12.
2. Bird, P.A., et al., *Intratympanic versus intravenous delivery of dexamethasone and dexamethasone sodium phosphate to cochlear perilymph.* Otol Neurotol, 2011. **32**(6): p. 933-6.
3. Rajan, G.P., et al., *The role of preoperative, intratympanic glucocorticoids for hearing preservation in cochlear implantation: a prospective clinical study.* Laryngoscope, 2012. **122**(1): p. 190-5.
4. van de Water, T.R., et al., *Mechanisms of hearing loss from trauma and inflammation: otoprotective therapies from the laboratory to the clinic.* Acta Otolaryngol, 2010. **130**(3): p. 308-11.
5. Wilk, M., et al., *Impedance Changes and Fibrous Tissue Growth after Cochlear Implantation Are Correlated and Can Be Reduced Using a Dexamethasone Eluting Electrode.* PLoS One, 2016. **11**(2): p. e0147552.
6. O'Connell, B.P., J.B. Hunter, and D.S. Haynes, *Current concepts in the management of idiopathic sudden sensorineural hearing loss.* Curr Opin Otolaryngol Head Neck Surg, 2016. **24**(5): p. 413-9.
7. Honeder, C., et al., *Sustained release of triamcinolone acetonide from an intratympanically applied hydrogel designed for the delivery of high glucocorticoid doses.* Audiol Neurootol, 2014. **19**(3): p. 193-202.
8. Oertel, R., W. Kirch, and E. Klemm, *Prednisolone concentration in the cochlea of patients with perilymph fistula.* Pharmazie, 2007. **62**(3): p. 239-40.
9. Niedermeyer, H.P., et al., *Cortisol levels in the human perilymph after intravenous administration of prednisolone.* Audiol Neurootol, 2003. **8**(6): p. 316-21.
10. Bird, P.A., et al., *Intratympanic versus intravenous delivery of methylprednisolone to cochlear perilymph.* Otol Neurotol, 2007. **28**(8): p. 1124-30.
11. Guzman, J., et al., *Triamcinolone acetonide protects auditory hair cells from 4-hydroxy-2,3-nonenal (HNE) ototoxicity in vitro.* Acta Otolaryngol, 2006. **126**(7): p. 685-90.
12. Kiefer, J., et al., *Conservation of low-frequency hearing in cochlear implantation.* Acta Otolaryngol, 2004. **124**(3): p. 272-80.
13. Cesar, I.C., et al., *Determination of triamcinolone in human plasma by a sensitive HPLC-ESI-MS/MS method: application for a pharmacokinetic study using nasal spray formulation.* J Mass Spectrom, 2011. **46**(3): p. 320-6.
