# Supplementary material for: Evaluation of Levels of Triamcinolone Acetonide in Human Perilymph and Plasma After Intratympanic Application in Patients Receiving Cochlear Implants: A Randomized Clinical Trial
Source: JAMA Otolaryngol Head Neck Surg. 2021 Sep 30;147(11):1–7. doi: 10.1001/jamaoto.2021.2492 (PMC8485207; doi:10.1001/jamaoto.2021.2492)
Supplement: Supplement 2. — Data Sharing Statement [file jamaotolaryngolheadnecksurg-e212492-s002.pdf]

Data Sharing Statement

Dahm. Evaluation of Levels of Triamcinolone Acetonide in Human Perilymph and Plasma After Intratympanic Application in Patients Receiving Cochlear Implants. *JAMA Otolaryngol Head Neck Surg*. Published September 30, 2021. doi:10.1001/jamaoto.2021.2492

Data
Data available: Yes
Data types: Deidentified participant data
How to access data: Data will be made available on reasonable request to the corresponding author - christoph.arnoldner@meduniwien.ac.at
When available: With publication

Supporting Documents
Document types: None

Additional Information
Who can access the data: Data will be made available on reasonable request to the corresponding author - christoph.arnoldner@meduniwien.ac.at
Types of analyses: For any purpose
Mechanisms of data availability: with a signed data access agreement
